# Circulating irisin levels in functional hypothalamic amenorrhea: a new bone damage index? A pilot study

**DOI:** 10.1007/s12020-022-03050-7

**Published:** 2022-04-15

**Authors:** Giovanna Notaristefano, Annamaria Merola, Elisa Scarinci, Nicolò Ubaldi, Monia Ranalli, Anna Tropea, Alice Diterlizzi, Simone Michele Fabozzi, Ornella Alesiani, Andrea Silvestrini, Alvaro Mordente, Esmeralda Capristo, Antonio Lanzone, Rosanna Apa

**Affiliations:** 1 Fondazione Policlinico Universitario A. Gemelli, IRCCS, L.go Agostino Gemelli, 8, 00168 Rome, Italy; 2 Università Cattolica del Sacro Cuore, Largo F. Vito 1, 00168 Rome, Italy; 3 Department of Statistical Sciences, Sapienza University of Rome, 00185 Rome, Italy

**Keywords:** Amenorrhea, Energy metabolism, Osteoporosis, Body composition, Bone diseases

## Abstract

**Purpose:**

Patients with functional hypothalamic amenorrhea (FHA) could commonly have bone damage, often preceded by metabolic alterations due to a relative energy deficit state. To date, there are no markers capable of predicting osteopenia before it is manifested on DXA. Irisin is a myokine that promotes the differentiation of osteoblastic cells and appears to be inversely correlated with the incidence of bone fragility and fractures in postmenopausal women. The aim of this study was to measure irisin levels in FHA patients and to correlate it with bone density parameters.

**Methods:**

Thirty-two patients with FHA and 19 matched controls underwent the same clinical and laboratory evaluation.

**Results:**

Irisin and body mass index (BMI) were significantly lower in the case group than in healthy controls (2.03 ± 0.12 vs. 2.42 ± 0.09 *p* < 0.05 and 19.43 ± 2.26 vs. 22.72 ± 0.67 *p* < 0.05, respectively). Additionally, total body mass density (BMD g/cm^2^) was significantly lower in the case group than in the healthy controls (1.09 ± 0.08 vs. 1.14 ± 0.05, *p* < 0.05), without signs of osteopenia.

**Conclusions:**

The FHA group showed lower irisin levels associated with significantly reduced BMD parameters that did not reach the severity of osteopenia. Therefore, we could speculate that irisin could predict DXA results in assessing modifications of body composition parameters. Future research is warranted to study these parameters in a larger population to confirm our results, so that irisin could be used as a predictor and screening method for bone deprivation. Furthermore, irisin is strictly related to energy metabolism and could be an indirect marker of nutritional status in FHA patients, identifying earlier states of energy deficit.

## Introduction

Functional hypothalamic amenorrhea (FHA) is defined as the absence or cessation of menstrual cycles due to the suppression of the hypothalamus-pituitary-ovary axis with GnRH pulsatility deficiency, without any associated organic injury. FHA can be related to stress, anxiety, weight change, energy imbalance, and/or excessive exercise. This clinical condition, although potentially reversible, is characterized by estrogen deficiency that can lead over time to the risk of developing osteopenia or osteoporosis. In young women, estrogens are the critical determinants which ensure proper bone metabolism acting the activation of bone remodeling units, the suppression of bone resorption and the stimulation of bone formation [1]. A 6 months lasting amenorrhea is an indication to a baseline dual-energy X-ray absorptiometry (DXA) evaluation of spine and hip bone density in any adolescent or woman with FHA [2]. The spine is the most common site of low bone density in adolescents and young women with amenorrhea and also predicts fracture risk. In adolescents older than 15 years of age and women with FHA, measuring hip bone density affords additional information about weight-bearing cortical bone and can be useful to monitor bone density longitudinally [3]. Hormonal and nutritional factors contribute 40–60% in constituting peak bone mass (PBM) [1]. An energy deficit (which can occur independent of changes in body weight) appears to be the critical factor in both the weight loss and exercise-induced forms of FHA. The coexistence of low energy availability (with or without eating disorders), amenorrhea/oligomenorrhea and reduced bone mineral density defines the so-called “female athlete triad” syndrome [4]. Many athletes are in a relative energy deficit state, as suggested by the presence of lower fat mass and hypothalamic amenorrhea. Nevertheless, nonathletes women with FHA are notoriously energy deprived too. They range from those who inadvertently or knowingly consume insufficient calories to match their caloric expenditure to those who have eating disorders and are severely undernourished. Such women therefore range in weight from normal to anorexic. Among these young women, bone density may be normal to low [2]. Osteopenia and osteoporosis are the main long-term complications of FHA. The goal of DXA is to identify individuals at risk for skeletal fragility, determine the magnitude of compromised bone mass in patients with established bone fragility, and guide and monitor treatment [3]. To date, there are no markers capable of predicting a bone damage before it is manifested at DXA.

Irisin is a hormone-like myokine involved in the conversion of white adipose tissue into brown adipose tissue (BAT) [5], thermogenesis (i.e., it affects the expression of thermogenin) [6] and energy homeostasis [7]. It is expressed in abundance in skeletal muscle in response to physical exercise. It comes from the proteolytic cleavage of FNDC5 (fibronectin type III domain-containing protein 5), a membrane protein that is highly expressed in skeletal muscle, heart, adipose tissue and liver [6].

In rodent models, irisin increases thne trabecular and cortical thickness and trabecular density enhancing osteoblast activation and RANKL-mediated inhibition of osteoclastogenesis [8]. In humans, this mechanism was confirmed [9]. Irisin has been found to be inversely correlated with serum sclerostin, an inhibitor of bone formation [10]. Additionally, by stimulating osteoblastogenesis, irisin is inversely correlated with the incidence of bone fragility and fractures in postmenopausal women [11], so it has been proposed as a potential marker for monitoring osteoporosis.

Concerning patients with FHA, lower irisin levels were found in athletes with amenorrhea than in athletes without amenorrhea and nonathletes [12].

The aim of our study was to analyse irisin levels in women with FHA, removing the confounding factor of physical exercise.

## Materials and methods

This was a monocentric observational retrospective study. The study protocol was approved by the Ethics Committee of Fondazione Policlinico Universitario A. Gemelli IRCCS (Prot N. 0018288/21 May 19, 2021; ID 3956), and all participants were enrolled only after receiving an explanation of the purpose and nature of the study. It was conducted in accordance with the Declaration of Helsinki, as revised in 2013. Informed written consent was obtained from all subjects.

Thirty-two patients with FHA and nineteen healthy controls were recruited from the Department of Women’s Health Sciences, of the Child and Public Health of Fondazione Policlinico Universitario A. Gemelli IRCCS. At the time of recruitment, patients were selected according to the inclusion criteria and were subjected to a full clinical evaluation, which included routine tests to assess their demographic, clinical, instrumental and laboratory characteristics.

The inclusion criteria were as follows: age 18–34 years old and a diagnosis of functional hypothalamic amenorrhea. The diagnosis of hypothalamic amenorrhea was made based on an absence of menstrual cycles for at least three months [2], with a negative MAP (medroxyprogesterone acetate) test and circulating values of estrogen less than 50 pg/ml and FSH and LH less than 10 mIU/ml, after excluding other anatomical-functional pathologies. The exclusion criteria for all participants were: oral contraceptives taken during the previous three months; physical activity for more than two hours per week; autoimmune diseases; anorexia nervosa; eating disorders; coexisting polycystic ovary syndrome; diabetes mellitus; major surgery in the last 3 months; and other hormonal dysfunctions (hypothalamic, pituitary, thyroidal or adrenal causes).

Healthy controls were enrolled among patients aged between 18 and 34 years attending the department. They were patients not affected by functional hypothalamic amenorrhea, with 10–12 menstrual cycles per year and patients performing less than 3 h of physical exercise per week. The exclusion criteria for the healthy controls were: diagnosis of functional hypothalamic amenorrhea, polycystic ovary syndrome and other hormonal dysfunctions (hypothalamic, pituitary, thyroidal or adrenal causes); oral contraceptives taken during the previous three months; physical activity for more than two hours per week; autoimmune diseases; anorexia nervosa; eating disorders; diabetes mellitus; major surgery in the last 3 months.

The subjects did not alter their dietary habits or lifestyle in the days preceding the visit. All patients underwent a clinical examination, which included ultrasonography. Furthermore, the patient underwent an evaluation of clinical nutrition: anthropometric evaluation; bioimpedance; handgrip strength test; administration of a Dietary Habits Analysis Questionnaire (FFQ); and classification of nutritional status according to the ESPEN-GLIM definition of malnutrition. BMI was calculated as the ratio of body weight (kg) to height (m^2^). Body composition was performed by using dual-energy X-ray absorptiometry (DXA), using a “whole body” densitometer (Lunar iDXA, GE Healthcare, Chicago, IL, USA) that allows, by means of 3 different scans, the determination of both the amount of lean mass and fat mass content and the bone mineral density. The source emits X-rays at 2 different energies (low and high), for example, 100 keV and 140 keV, and the scanner analyses the amount of X-rays that pass through the body tissue in each pixel smaller than 1 mm^2^. The associated software calculates for each pixel the corresponding area of the bone, fat and lean components (muscle mass) using attenuation equations. The dose of the radiation administered was very low, with an anode current of less than 5 ma. Patients were given a questionnaire about daily physical activity (IPAQ: International Physical Activity Questionnaire), which measures the type and amount of physical activity performed. The questions also refer to the activity done in the last 7 days at work, to move from one place to another and during their leisure time.

On the same day, after fasting overnight for 10–12 h, blood samples were collected for hormonal assays: estradiol (E2), follicle-stimulating hormone (FSH), luteinizing hormone (LH). A second venous blood sample was obtained to evaluate the circulating irisin levels.

### Biochemical measurements

All hormonal assays were performed by electrochemiluminescence immunoassay kits (Roche Diagnostics, Mannheim, Germany). The intra- and interassay coefficients of variation for all of the hormones were < 7%.

Circulating irisin levels were quantified in the plasma samples using a specific immunoenzymatic ELISA kit (Cat. No. EK-067-029 from Phoenix Pharmaceuticals, Karlsruhe, Germany), which was previously validated by mass spectrometry analysis [13]. Intra- and interassay variations were below 10% and 15%, respectively, and the detection limit was 0.1 ng/ml. The optical density at 450 nm was measured, with a reading time of 1 s, using a microplate reader (Victor3; Perkin Elmer, USA) with 0.5% precision at 450 nm and the temperature control set to 25 °C. The analyses were performed in duplicate.

### Statistical analysis

All of the results were obtained using R statistical software (free open source).

The following statistical methodologies were used to verify and empirically support the study objectives. Irisin levels were logarithmically transformed prior to the analyses to approximate a normal distribution. The correlation of the irisin levels with bone density (DXA), lean mass (kg), fat mass (kg) and BMI in patients with FHA was first quantified from a descriptive point of view through the correlation matrix. This matrix indicated both the direction (positive or negative) and the intensity of the dependence (strong or weak) between the variables. Subsequently, to investigate the statistical significance of these possible relationships, a linear regression model was implemented whose dependent variable was irisin, while DXA, fat mass (kg), lean mass (kg) and BMI were the main regressors. This allowed us both to verify the significance of the regression and to quantify the marginal increase or decrease of the level of irisin against a unit increase of a variable (DXA, fat mass (kg), lean mass (kg) or BMI), keeping the remaining variables constant.

To support and verify empirically whether basal plasma irisin levels in FHA patients may be significantly different from the control group levels, namely, healthy patients, a t-test for independent groups was conducted. Since the variable under study is usually asymmetric, a logarithmic transformation was applied before the test was conducted, and then it was possible to assess whether the difference between the average levels in the two groups could be considered statistically significant. A bilateral t-test was conducted assuming that the variances in the two groups were equal. The choice of the alternative hypothesis did not affect the statistical methodology used. In fact, a bilateral test is more conservative and therefore requires a higher sample size, with an equal power and significance level, compared to a one-sided test. The system of assumptions therefore consisted of the same averages under the null hypothesis and different averages under the alternative hypothesis. It rejected the null hypothesis; that is, if the p value was less than 0.05, the difference between averages was statistically significant.

## Results

The clinical characteristics of both the FHA patients and the controls, with particular attention to the risk factors for the functional hypothalamic amenorrhea (such as BMI, lean and fat mass index, bone density, physical activity habits, etc.) are presented in Table 1 as the mean and standard deviation (SD).

**Table 1:** Clinical characteristics of women with functional hypothalamic amenorrhea (FHA group) versus clinical characteristics of healthy patients (controls)

	FHA group		Controls		FHA group vs. Controls		
Variable	Mean	St. Dev	Mean	St. Dev	*t*-value	*p*-value	95% Confidence interval
Irisin	7.67	0.93	11.28	1.04			
Log Irisin	2.03	0.12	2.42	0.09	−13.024	0.00000*	(−0.448, −0.328)
Height (m)	1.64	0.06	1.64	0.04			
Weight (kg)	52.61	6.95	61.14	3.70			
BMI (kg/m^2^)	19.43	2.26	22.72	0.67	−7.871	0.00000*	(−4.132, −2.440)
E2 (pg/ml)	34.39	13.78	55	27.34	−2.817	0.011*	(−35.894, −5,320)
Fat mass (kg)	13.85	4.00	17.03	3.18	−3.482	0.00095*	(−5.012, 1.353)
Lean mass (kg)	38.44	3.76	38.86	4.45	−0.408	0.6848	(−2.529,1.673)
Body fat (%)	25.82	5.54	27.70	3.83	−1.566	0.1232	(−4.296, 0.528)
Total BMD (g/cm^2^)	1.09	0.08	1.14	0.05	−2.911	0.005231*	(−0.087, 0.016)
Spine BMD (g/cm^2^)	1.28	0.17	1.22	0.09	1.850	0.07059	(−0.006, 0.133)
Spine BMD Z scores	0.76	0.85	0.25	0.13	3.319	0.002236*	(0.195, 0.815)
Total hip BMD (g/cm^2^)	1.09	0.18	1.15	0.10	−1.667	0.1017	(−0.136, 0.013)
Total hip BMD Z scores	0.47	0.55	0.38	0.19	0.860	0.3951	(−0.120, 0.298)
Femoral neck BMD (g/cm^2^)	1.04	0.09	1.10	0.08	2.831	0.00631*	(−0.107, −0.018)
Femoral neck BMD Z scores	0.41	0.49	0.30	0.15	1.168	0.2512	(−0.082, 0.303)

Bold values identify statistical significance

^*^ *p* value < 0.05

*BMI* Body mass index, *E2* Estradiol, *BMD* Bone mineral density

Patients in the study group suffered a mean period of amenorrhea of 8 months. The majority of women in the study group developed amenorrhea secondary to weight loss. The mean weight reported in the study group was 52.61 ± 6.95 kg, whilst in the control group it was 61.14 ± 3.70 kg. The main clinical features for both the cases and controls are summarized in Table 1. Concerning the control group, their clinical variables were more homogenous than those of the FHA group.

**Fig. 1:**
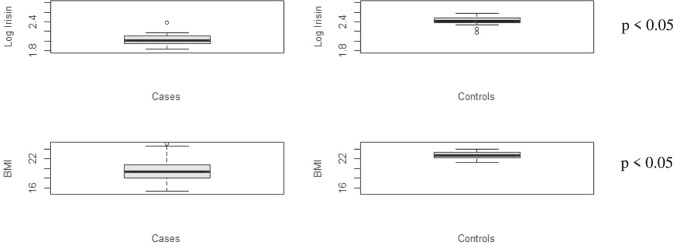
Boxplots of Log Irisin and BMI distributions over the case and control groups

Irisin and BMI were significantly lower in the FHA group than in the healthy controls. Looking at Fig. 1, the distributions of the two variables (log irisin and BMI) over the groups seemed to be different. To assess whether they were significantly different, we conducted a two-tailed t-test assuming equal variances in the two populations at a significance level of 0.05 with 95% confidence intervals. As shown in Table 1 their means were significantly different. The other variables significantly different between the two groups were: estrogen (*p* = 0.011), fat mass (kg) (*p* = 0.0009), total BMD (g/cm^2^) (*p* = 0.0052), spine BMD Z scores (*p* = 0.0022) and femoral neck BMD (g/cm^2^) (*p* = 0.0063).

Finally, we noted that the mean femoral neck BMD Z scores were higher in the FHA group than in the controls; furthermore, the distribution in the FHA was more heterogeneous (higher SD). However, it is worth noting that the two means were not significantly different (*p*-value = 0.2512).

To exclude the influence of vitamin D, parathormone (PTH) and FSH on BMD, we conducted the simple linear regressions between total BMD, spine BMD, spine BMD z-score, total hip, total hip z-score, femoral neck and femoral neck z-scores. All the regressions were not significant apart spine BMD z scores versus vitamin D (*p* = 0.028). However, the effect of vitamin D on spine BMD z score is almost null; for each unit increase of vitamin D, then spine BMD z score increases by 0.04. Furthermore, since all the other regressions were not significant, it seems that vitamin D, as well as, parathormone and FSH, do not have a direct effect on BMD.

Furthermore, we conducted three simple linear regression models between the irisin and vitamin D, parathormone and FSH, respectively. The regressors are not significant as shown in Table 2.

**Table 2:** Single linear regression between log irisin and vitamin D, parathormone (PTH), and FSH

	Estimate	Std. Error	t value	*p*-value
Intercept	2.0735	0.0851	24.375	<2e-16*
Vitamin D	−0.0013	0.0023	−0.543	0.592
Intercept	2.0059	0.0710	28.26	<2e-16*
PTH	0.0005	0.0014	0.33	0.744
Intercept	2.0246	0.0927	21.833	<2e-16*
FSH	0.0006	0.0137	0.043	0.966

^*^ *p* < 0.05

Furthermore, we analysed the dependence among the variables in the FHA group. We have fitted several linear regression models, both singular and multiple, considering as response variables log irisin and as the sets of regressors BMI, fat mass, lean mass, body mass (%), total BMD, spine BMD, total hip BMD, femoral neck BMD, spine BMD Z scores, total hip BMD Z scores, and femoral neck BMD Z scores. Two observations were found to be influential. The following results were obtained through a robust regression analysis using the function lmRob in the package robust. We obtained 7 significant linear regression models, of which 2 were singular and 5 were multiple. For the multiple regression, the variable selection was based on the backwards selection procedure.

**Fig. 2:**
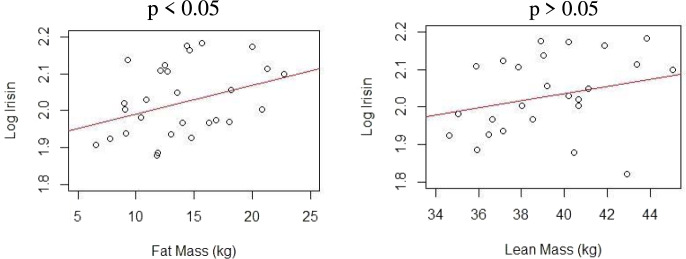
Single linear regression between log Irisin versus fat mass and lean mass

Concerning the single linear regression analysis, irisin was positively associated with fat mass and lean mass content, separately, as shown in Table 3. By increasing 1 unit of fat mass and lean mass, irisin will increase by 0.78% (that is, [exp(0.0078)-1]*100) and 0.94% (that is, [exp(0.0094)-1]*100), respectively (Fig. 2). The fitted models were able to explain 10.27% and 11.26%, respectively, of the variability of log irisin.

**Table 3:** Single linear regression between log irisin and fat mass and between log irisin and lean mass

	Estimate	Std. Error	t value	*p* value
Intercept	1.9120	0.0654	29.246	<2e-16*
Fat mass (kg)	0.0078	0.0045	1.718	0.0961
Residual standard error: 0.1027 on 30 degrees of freedom Multiple R-squared: 0.1027				
Intercept	1.6592	0.1745	9.508	<1.46e-10*
Lean mass (kg)	0.0094	0.0045	2.076	0.0465*
Residual standard error: 0.09866 on 30 degrees of freedom Multiple R-squared: 0.1126				

Bold values identify statistical significance

^*^ *p*-value < 0.05

Table 4 shows the significant association between irisin and lean mass content, spine BMD, femoral neck BMD, total hip BMD, spine BMD Z scores, femoral neck BMD Z scores and total hip BMD Z scores.

**Table 4:** Multiple linear regression between log irisin versus lean mass, spine BMD, femoral neck BMD, total hip BMD, spine BMD Z scores, femoral neck BMD Z scores, and total hip BMD Z scores

	Estimate	Std. Error	*t*-value	*p*-value
Intercept	2.3124	0.2858	8.092	6.86e-08*
Lean mass (kg)	−0.0116	0.0047	−2.494	0.0210*
Spine BMD (g/cm^2^)	1.2858	0.1958	6.568	1.67e-06*
Femoral neck BMD (g/cm^2^)	−1.7672	0.3106	−5.690	1.20e-05*
Total hip BMD (g/cm^2^)	0.5574	0.0956	5.833	8.63e-06*
Spine BMD Z scores	−0.0544	0.0274	−1.985	0.0604
Femoral neck BMD Z scores	−0.4868	0.0895	−5.441	2.13e-05*
Total hip BMD Z scores	0.1383	0.0598	2.311	0.0311*

Bold values identify statistical significance

^*^ *p*-value < 0.05

Residual standard error: 0.05853 on 21 degrees of freedom Multiple R-squared: 0.3408

*BMD* Bone mineral density

By increasing 1 unit of spine BMD, total hip BMD and total hip Z scores will cause irisin to increase by 261.76% (that is, [exp(1.2858)-1]*100), 74.61% (that is, [exp(0.5574)-1]*100) and 14.83% (that is, [exp(0.1383)-1]*100), respectively. On the other hand, by increasing 1 unit of lean mass, femoral neck BMD, spine BMD Z scores and femoral neck BMD Z scores will cause irisin to decrease by 1.15% (that is, [exp(-0.0116)-1]*100), 82.92% (that is, [exp(−1.7672)-1]*100), 5.29% (that is, [exp(-0.0544)-1]*100) and 38.54% (that is, [exp(-0.4868)-1]*100), respectively. The fitted model was able to explain 34.08% of the variability of log irisin.

**Fig. 3:**
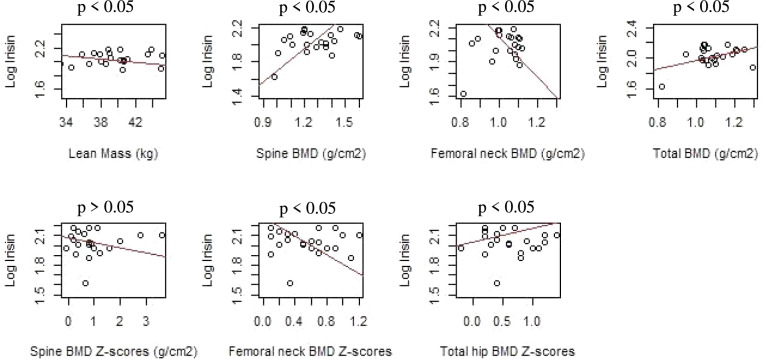
Effect of each regressor on the log-irisin

The effect of each variable on the log irisin while keeping the others fixed is plotted in Fig. 3. The observations were weighted according to the weights of the regression.

It is worth noting that in the multiple regression analysis, the effect of the singular predictor on irisin was statistically significant only if the other predictors were also present. It can be inferred that the dependence (correlation) between the predictors had a stronger effect on irisin. In other words, the effect on irisin cannot be explained only by a single variable.

We observed that different subsets of variables were significantly associated with irisin, even if their single effect was different due to the dependence on the other variables included in the model. Indeed, the same regressor could cause a different change in the irisin values if the model included a different set of regressors.

## Discussion

To date, this is the first study in the literature to demonstrate low irisin levels in nonathletes and nonanorexic women suffering from FHA. Irisin levels were found to be significantly lower in the FHA group than in healthy controls.

It is known that patients with FHA commonly have a decreased Body Mass Index (BMI) value, as well as a reduced lean and fat mass content and reduced bone density. Irisin is related to parameters such as energy balance, lean and fat mass content and bone density [14] and the same parameters are disrupted in FHA patients. Women affected by FHA enter a “survival mode” due to the inhibition of regular GnRH pulsatility, which normally allows for correct functioning of the HPO (hypothalamus-pituitary-ovary) axis and consequently reproduction. To date, only one study has been published concerning the association of irisin and FHA patients [12]. However, it only examined female athletes. The authors concluded that irisin levels were lower in athletes with amenorrhea than in athletes without amenorrhea and nonathletes and these differences persisted after controlling for measures of body composition. In our study, we considered physical exercise and eating disorders as confounding factors because they affect irisin levels independently [15], and by excluding their influence, we could highlight the impact of psychogenic stress and its consequent energy deprivation in patients with FHA. Under conditions of normal energy homeostasis, irisin promotes the “browning” of white adipose tissue [16], thus favouring thermogenesis and energy expenditure. Therefore, in this study we confirmed the hypothesis that irisin levels are lower in FHA patients than in controls as a defensive mechanism to restrict brown adipose tissue (BAT) conversion [17]. BAT has an inherent capacity to convert excess energy into thermal energy [18]. States of severe energy deficit, such as anorexia nervosa, are associated with reduced BAT. BAT’s fundamental role is to dissipate energy in the form of heat by converting its molecular structure from white adipose tissue as a defensive mechanism against cold. Regular physical activity normally causes an increase in BAT, which potentially contributes to the increase in resting energy expenditure (REE) and caloric expenditure observed in athletes [4]. It is important to consider that REE represents 70% of the total energy expenditure [19] and is strictly related to lean body mass. Nevertheless, in elite athletes, BAT activity may be reduced as an adaptive response to conserve energy and to reduce REE, thus preventing the conversion of white adipose tissue to brown, as has been reported in endurance athletes [20]. In athletes, irisin levels were positively associated with resting energy expenditure (REE), reflecting the effects of chronic exercise on irisin levels [12]. Interestingly, current scientific data do not support the concept of irisin being induced by exercise under conditions of extremely low body weight, such as anorexia nervosa [21]. We can speculate that to avoid further deprivation, our body enters a defensive mechanism that restricts BAT conversion to both preserve a state of well-being and prevent further unsustainability. Therefore, our data seem to confirm the hypothesis that irisin is reduced in energy deficit states as a defensive mechanism against further energy expenditure, thus preventing the conversion of white to brown adipose tissue. This is supported by findings of lower irisin levels in anorexic patients [17] and in patients who have lost large amounts of weight after having undergone bariatric surgery [22].

Consistent with other studies, in our study, irisin levels had a positive association with fat mass and lean mass; as these parameters were reduced, circulating irisin levels also decreased. It is pivotal to emphasize the fact that the FHA group showed a lower BMI than the healthy controls but it was still within the normal range, in addition to a lower fat mass and body fat percentage. Interestingly, lean mass content was similar between the two groups, unlike what happens in extremely low body weight conditions. Previous studies that investigated irisin in anorexia nervosa enlisted patients with BMIs of 12.5 and 14.2 kg/m^2,^ respectively, as long as they presented with eating disorders such as binge eating or purging [17, 23].

Low irisin levels could contribute to the transition towards osteopenia and osteoporosis as seen in adult women [24]. In addition, irisin has been reported to have a beneficial role in bone metabolism by promoting osteoblast proliferation and differentiation [9]. In the present study, the FHA group showed lower bone mineral density (BMD) parameters, without ever reaching levels of osteopenia. Furthermore, irisin levels were positively associated with bone density parameters; however, each of them did not affect irisin independently. Patients affected by FHA have reduced BMD mainly due to an estrogen deficit condition that over the time increases the risk of developing osteopenia or osteoporosis. Nevertheless, in addition to estrogen, irisin depletion may contribute to the reduced trabecular and cortical thickness and trabecular density found in these patients. Since it has been reported in vitro that irisin promotes osteoblastic cells differentiation [8], a reduced value of irisin concentration could contribute to decreased bone density. The effects of irisin on the bone may also be mediated via the induction of brown adipogenesis, given the reports of associations of brown fat with bone strength estimates in women. Additional evidence has highlighted how BAT could contribute to promoting the differentiation of the osteoblastic lineage. Indeed, brown adipocytes have the potential to differentiate into either osteoblasts or myocytes [25]. It has been shown that anorexic women with active BAT had increased bone density levels compared to controls [26]. Therefore, BAT is an independent predictor of bone density in women [27] and both BAT volume and activity are positively associated with total and cortical bone cross-sections in children and adolescents [25].

In the present study BAT was not measured; hence, this could be an area for future studies. In addition, we did not measure REE in the two groups, although we know it is positively associated with irisin [28]. The average amenorrhea period considered was quite short (eight months). Most likely, a longer period of amenorrhea would lead to even more significant changes in the bone that were associated with irisin levels.

## Conclusions

The present study reports reduced irisin levels in nonathletes and nonanorexic patients affected by FHA. The FHA group showed lower irisin levels associated with significantly reduced BMD parameters that did not reach the severity of osteopenia. Therefore, we could speculate that irisin could predict DXA results in assessing modifications of body composition parameters. The prediction of a possible bone damage would allow targeted therapies to be performed earlier, to avoid a condition of permanent osteopenia or osteoporosis. Future research is warranted to study these parameters in a larger population to confirm our results, so that irisin could be used as a predictor and screening method for bone deprivation. Furthermore, irisin is strictly related to energy metabolism and could be an indirect marker of nutritional status in FHA patients, identifying earlier states of energy deficit.

## Data Availability

The datasets generated during and/or analysed during the current study are available from the corresponding author on reasonable request.
